# Supplementary material for: Complexome profiling of the Chlamydomonas psb28 mutant reveals TEF5 as an early PSII assembly factor
Source: Plant Cell. 2025 Mar 17;37(6):koaf055. doi: 10.1093/plcell/koaf055 (PMC12164590; doi:10.1093/plcell/koaf055)
Supplement: koaf055_Supplementary_Data [file koaf055_supplementary_data.zip › psb28_interactive.zip!/plots/intensity/qualityPlots/peptides/_Cre06.g275100.t1.2.html]

### Cre06.g275100.t1.2

Trivial name:   
SeqCoverage [median]: 15.80  
Mass: 53.64 kDa  
  
 

| Sequence | MissedCleavage | Mass [Da] | wtIntensity | muIntensity | Score |
| --- | --- | --- | --- | --- | --- |
| AGEYDGSEVAGGYIK | 0 | 1514.69 | 0.00 | 1067.90 | 92.87 |
| AGSELGGR | 0 | 745.37 | 1622.55 | 3391.00 | 120.62 |
| IGMDAATGR | 0 | 890.43 | 2060.18 | 4158.84 | 163.20 |
| KADDDEEEEAEEEEPVIK | 1 | 2102.90 | 0.00 | 481.24 | 79.97 |
| NIKVEVAQAR | 1 | 1126.65 | 2568.65 | 4706.01 | 162.80 |
| NKAGEYDGSEVAGGYIK | 1 | 1756.83 | 0.00 | 635.07 | 46.62 |
| VDVNVQR | 0 | 828.45 | 0.00 | 2600.59 | 149.40 |
| VEVAQAR | 0 | 771.42 | 0.00 | 917.68 | 91.91 |
|  |  |  | 6251.4 | 17958.3 |  |
